# Global perspective of environmental distribution and diversity of Perkinsea (Alveolata) explored by a meta-analysis of eDNA surveys

**DOI:** 10.1038/s41598-023-47378-0

**Published:** 2023-11-17

**Authors:** Sebastian Metz, Sarah Itoïz, Aleix Obiol, Evelyne Derelle, Ramon Massana, Cédric Berney, Colomban de Vargas, Philippe Soudant, Adam Monier, Aurélie Chambouvet

**Affiliations:** 1 Sorbonne Université, CNRS, UMR7144 Adaptation et Diversité en Milieu Marin, Ecology of Marine Plankton (ECOMAP), Sorbonne Universités, Station Biologique de Roscoff, Place Georges Teissier, 29680 Roscoff, France; 2 CNRS, IRD, Ifremer, LEMAR, Univ Brest, Plouzané, France; 3 Rivages Pro Tech, 2, Allée Théodore Monod, 64210 Bidart, France; 4 Department of Marine Biology and Oceanography, Institut de Ciències del Mar (ICM-CSIC), Barcelona, Spain; 5 Living Systems Institute, University of Exeter, Stocker Road, Exeter, UK; 6 Present Address: Department of Archaeology, University of York, York, UK

**Keywords:** Ocean sciences, Marine biology, Microbiology, Microbial communities, Environmental microbiology, Parasitology, Pathogens, Ecology, Biodiversity

## Abstract

Perkinsea constitutes a lineage within the Alveolata eukaryotic superphylum, mainly composed of parasitic organisms. Some described species represent significant ecological and economic threats due to their invasive ability and pathogenicity, which can lead to mortality events. However, the genetic diversity of these described species is just the tip of the iceberg. Environmental surveys targeting this lineage are still scarce and mainly limited to the Northern Hemisphere. Here, we aim to conduct an in depth exploration of the Perkinsea group, uncovering the diversity across a variety of environments, including those beyond freshwater and marine ecosystems. We seek to identify and describe putative novel organisms based on their genetic signatures. In this study, we conducted an extensive analysis of a metabarcoding dataset, focusing on the V4 region of the 18S rRNA gene (the EukBank dataset), to investigate the diversity, distribution and environmental preferences of the Perkinsea. Our results reveal a remarkable diversity within the Perkinsea, with 1568 Amplicon Sequence Variants (ASVs) identified across thousands of environmental samples. Surprisingly, we showed a substantial diversity of Perkinsea within soil samples (269 ASVs), challenging the previous assumption that this group is confined to marine and freshwater environments. In addition, we revealed that a notable proportion of Perkinsea ASVs (428 ASVs) could correspond to putative new organisms, encompassing the well-established taxonomic group Perkinsidae. Finally, our study shed light on previously unveiled taxonomic groups, including the Xcellidae, and revealed their environmental distribution. These findings demonstrate that Perkinsea exhibits far greater diversity than previously detected and surprisingly extends beyond marine and freshwater environments. The meta-analysis conducted in this study has unveiled the existence of previously unknown clusters within the Perkinsea lineage, solely identified based on their genetic signatures. Considering the ecological and economic importance of described Perkinsea species, these results suggest that Perkinsea may play a significant, yet previously unrecognized, role across a wide range of environments, spanning from soil environments to the abyssal zone of the open ocean with important implications for ecosystem functioning.

## Introduction

Climate change and human activities have become catalysts for spreading infectious diseases affecting humans and wildlife^1,2^. These factors contribute to the introduction of new pathogens to host populations that were previously unexposed^1^. While studies of exotic microbes have primarily focused on plant pathogens in terrestrial ecosystems for agricultural purposes^3^, extending this knowledge to other environments harboring vast amounts of unexplored microbial diversity is crucial. Among microeukaryotes, parasitism is widespread across various environments, including marine, freshwater and soil ecosystems^4–6^. Previous studies have emphasized the crucial role of parasites in energy flow, ecological organization, and ecosystem resilience^7^. However, the relationship between parasite diversity and host range remains unknown^7^. This knowledge gap is particularly concerning since some parasitic taxa could be important drivers of disease outbreaks.

The Alveolata supergroup comprises several taxonomic groups of microeukaryotic parasites, including the well-studied Apicomplexa group (e.g., the human pathogens *Plasmodium* and *Toxoplasma*), the Syndiniales, which include marine alveolate groups I and II (MALV-I and MALV-II), and the Perkinsea lineage (also known as Perkinsids or Perkinsozoa). Despite its socio-economic and ecological significance, the Perkinsea lineage has received limited attention in environmental surveys^8^. Only few lineages from aquatic ecosystems have been described so far^8^. Among them, the Perkinsidae group infects mollusks, which includes two well-known species, *Perkinsus olseni* and *Perkinsus marinus*. These are considered the most ecologically and economically destructive marine pathogens and are listed as notifiable diseases by the World Organization for Animal Health (O.I.E.)^9,10^. Parviluciferaceae infect a wide range of dinoflagellates, including toxic species, and play a crucial role in host species succession and red tide dynamics^11^. Two other marine parasites of dinoflagellates, *Maranthos nigrum* and *Pararosarium dinoexitiosum*, were recently described branching outside the Parviluciferaceae^12,13^. The Xcellidae lineage, a recently described group, parasitizes marine fishes^14–17^. Despite infecting vulnerable and commercially important fish species, the impact of these parasites on the host population dynamics remains undescribed^15,16^. Hence, most representative taxa of the Perkinsea lineages have been identified in marine environments, except for the Severe Perkinsea Infection (SPI) agent, a parasite of tadpoles^8^. The SPI agent can cause mortality rates of up to 95% in frog populations, making it an emerging threat to amphibians^18–22^. These organisms belong to a monophyletic group previously referred to as Novel Alveolate Group 01 (NAG01)^19^*.* Finally, although no genetic characterization has been performed so far, *Rastrimonas subtilis* has been described as a parasite of the freshwater *Chilomonas paramecium*^23,24^. This finding was supported by Jobard et al.^25^, who describe the association between freshwater Perkinsea with chlorophyte algae using fluorescent in situ hybridization (FISH). These results provide insights into the potential parasitic lifestyle of these enigmatic organisms in freshwater environments.

Environmental DNA (eDNA) studies, based on the sequencing of the small sub-unit ribosomal RNA gene (SSU rDNA), have unveiled high diversity of Perkinsea-like sequences, with the described lineages representing just the tip of the iceberg^8^. In freshwater ecosystems, Perkinsea sequences are abundant and form clusters exclusively composed of environmental sequences. This indicates the existence of unrecognized diversity that likely plays significant but overlooked ecological roles in the food web dynamics^25–27^. In marine environments, Perkinsea sequences account for less than 1% of the reads of DNA/RNA datasets^28^. However, they are widely distributed in coastal areas, especially in sediments, with a significant proportion of reads detected in RNA-derived samples. These results suggest metabolic activity of detected Perkinsea and potential participation in the ‘seed bank’ of microbial communities^28,29^. Nevertheless, these studies have limited geographical coverage, mainly focusing on specific geographical regions in the Northern Hemisphere^28^, giving only a partial view of the Perkinsea diversity and distribution.

In this paper, we conducted a comprehensive analysis of a metabarcoding dataset, which includes sequences of the V4 region of the SSU rRNA gene from diverse ecosystems, including marine, land water and soil environments (the EukBank dataset), with the aim to investigate the diversity, distribution, and environmental preferences of the Perkinsea lineage in a wide range of samples from land to aquatic ecosystems^8^. Overall, our results underscore the significance of incorporating large-scale eDNA surveys to gain a comprehensive understanding of overlooked microeukaryotic organisms that may be related to parasites implicated in disease outbreaks worldwide.

## Methods

### Perkinsea reference phylogenetic tree

We conducted a phylogenetic analysis of Perkinsea to classify the Amplicon Sequence Variants (ASVs) retrieved from the EukBank dataset (details provided in the following section). For the analysis, we selected 95 sequences that encompassed the known diversity of Perkinsea^8,19,25,28,30^ and seven sequences as outgroup references [*Ichthyodinium chabelardi* (AB264776), *Noctiluca scintillans* (AF022200), *Amoebophyra* sp. (AF472555), *Hematodinium perezi* (EF065717), *Symbiodinium microadriaticum* (M88521), *Amyloodinium ocellatum* (AF080096) and *Alexandrum ocellatum* (U27500)]. The sequences were aligned using MAFFT V 7.407^31^ with the *E-INS-I* algorithm and trimmed automatically with trimAl v1.4.rev15^32^, discarding regions with more than 75% gaps using a *-gt* parameter of 0.25. Maximum likelihood (ML) analysis was performed using RaxML-ng V1.1^33^ with the GTR + F + I + G4 model, determined as the best-fit model by ModelFinder^34^. The final ML tree was generated from 25 parsimonious and random trees. We reconstructed 1000 non-parametric bootstrap trees to evaluate node supports and incorporated support values into the ML tree using the Transfer Bootstrap Expectation support metric (TBE) metric^35^. Bayesian analysis was conducted using MrBayes v3.2.6^36^ with the GTR + G + I model (*lset*, *nst* = *6 rates* = *invgamma*) running two replicates of four Markov chain Monte Carlo chains for 2,500,00 generations, using *heat* parameter of 2. Trees were sampled every 100 generations and the initial 25% were discarded as burn-in. Consensus topologies and posterior probabilities of each node were then calculated based on the remaining trees. The reference tree (best-scoring ML) was annotated according to the bootstrap values (TBE > 80), posterior probabilistic values (PP > 0.9), and supporting literature^8,19,25,28,30^. Environmental clusters were classified as ‘Perkinsea cluster 01–04’, representing clusters composed solely of environmental sequences.

### The EukBank database and Perkinsea ASVs phylogenetic classification

The EukBank database is a compilation of eDNA surveys that employed high-throughput sequencing methods (Illumina MiSeq and Roche 454) targeting the hypervariable region V4 of the SSU rDNA sequence^37^. Briefly, raw sequences were obtained from the EMBL/EBI-ENA EukBank umbrella project. When applicable, reads were trimmed with Cutadapt (https://github.com/marcelm/cutadapt/^38^ with specific parameters tailored to extract fragments covered by the primers sets TAReuk454FWD1 and TAReukREV3 from the V4 region or the SSU rRNA gene^39^. Identical sequences were merged with VSEARCH^40^, followed by clustering with Swarm^41^. Subsequently, chimera detection was conducted using the *--uchime_denovo* function in VSEARCH^42^, and low-quality sequences were filtered out. The final set of ASVs was obtained based on occurrence patterns, utilizing a modified version of the Lulu algorithm^43^, which can be found at https://github.com/frederic-mahe/mumu. Taxonomic classification of the ASVs was performed using the curated EukRibo database version 1.0^44^, employing the global pairwise alignment approach (*--usearch_global* from VSEARCH). This database was generated by the UniEuk consortium^37^.

A phylogenetic placement method was applied to ASVs initially affiliated with Perkinsea^45,46^. The sequences were added to the reference alignment using MAFFT with the *--addfragments* and *--keeplength* parameters and then placed onto the reference tree using the evolutionary placement approach EPA-ng v0.3.8^46^. The results were subsequently analyzed using Gappa^47^. ASVs not affiliated with Perkinsea were discarded. These included ASVs placed within the outgroup or those with long branches that could not be confidently assigned through manual inspection using NCBI BLASTn with default parameters^48^.

### Diversity, distribution and environmental preferences of Perkinsea

To investigate the distribution and ecological preferences of the phylogenetic groups within Perkinsea, samples were classified into three main categories: ‘Marine’, ‘Land water’ and ‘Soil’. Marine samples were further sub-classified into ‘Epipelagic zone’ (0–200 depth meters), ‘Mesopelagic zone’ (200–1000 depth meters), ‘Bathypelagic zone’ (1000–4000 depth meters), ‘Abyssal zone’ (below 4000 depth meters), ‘Sediment’, ‘Pelagic’ (include marine water samples from unspecified depth), ‘Artic mixed water’, ‘Coastal zone’ (including sub-littoral, intertidal and grass bed of coastal seawater), ‘Estuarine’ and ‘Marine other’ (including marine biofouling and coral samples).

Land water samples were categorized as ‘Lakes’, ‘Rivers’, ‘Sediments’, ‘Brackish’, ‘Bromeliads tank water’, ‘High Arctic water’ and ‘Saline spring sediment’. ‘Soil’ samples were divided into ‘Cropland (Temperate/Tropical)’, ‘Forest (Temperate/Tropical)’, Grassland (Temperate) and ‘Land soil (Temperate)’.

To analyze the composition of Perkinsea diversity, a non-metric multidimensional scaling ordination plot (NMDS) based on the Bray–Curtis dissimilarity was conducted using the *vegan* package v2.5–7^49^ Samples with more than 10,000 reads and at least one Perkinsea ASV were extracted from the EukBank dataset and rarefied using the *rrarefy* function from *vegan* package. From each environmental type, 15 samples with the highest Shannon diversity were selected to create a subset of samples representing the highest observed diversity. This approach was adopted to avoid outliers, as many samples contained only one or a few ASVs with very low abundance. Subsequently, the samples were transformed using the Hellinger method^50^ with the *decostand* function, and the NMDS analysis was performed using the Bray–Curtis dissimilarity index with the *metaNMDS* function. To test the hypothesis of significant difference between the communities of the main categories and between environments, we used a non-parametric test of significant difference ANOSIM (ANalysis Of Similarities) with the function *anosim* from the *vegan* package and *pairwise.adonis* from *pairwiseAdonis* package^51^ using the parameters *sim.method* = *bray.*

For the community structure analysis based on phylogenetic distance, the Picante and Phyloseq R packages were used^52,53^. The alignment of the ASVs and the outgroup sequences, obtained from the previous Perkinsea ASVs phylogenetic classification, was extracted. The best-ML tree and the rarefy table were used to calculate the sample distance using the weighted UniFrac method. The same samples used in the NMDS analysis were also employed for a principal coordinates analysis (PCoA). Additionally, Faith’s Phylogenetic Diversity (PD)^54^ and the Nearest Relative Index (NRI)^55^ were calculated for each environment. The phylogenetic difference between the environments was tested with *pairwise.adonis* using the UniFrac weighted distance matrix.

To explore the global distribution of Perkinsea, the proportion of ASVs related to each taxonomic group observed in the different geographical regions was plotted on a world map. The environmental preferences or occurrences were investigated by calculating the number of ASVs observed in each environment, represented using a chord diagram created with the R package *circlize*^56^. To assess the overlap of ASVs between the ‘Marine’, ‘Land water’ and ‘Soil’ categories a Venn diagrams were generated using the R package *nVennR*^57^. All the analyses were conducted in R (R Core Team, 2020) and scripts for these analyses are available on GitHub (https://github.com/sebametz/perkinsea_distribution).

### ASVs related to potential novel Perkinsea organisms

To test the novelty of each ASV, two criteria were selected: the similarity of each ASV to its closest related sequence from a curated Perkinsea dataset of sequences obtained from NCBI and the likelihood weight ratio (LWR) derived from the placement of the ASVs into the reference phylogenetic tree. The LWR serves as an indicator of the confidence in the placement of the ASVs within the phylogenetic tree^47^. A low LWR value suggests a less reliable representation of the ASV’s taxonomy in the phylogeny. Since our phylogeny aimed to encompass the known diversity of Perkinsea, ASVs with low similarity to reference sequences from the database and low LWR are likely associated with potential novel organisms whose taxonomy is unknown. We classified an ASV as a novel if its similarity percentage was below the mean similarity with the reference dataset (< 94.8%) and if its LWR was below the mean observed for the placement of all Perkinsea ASVs (< 0.69). The environmental distribution of the novel ASV was investigated using a combination of their phylogenetic placement and the number of observations of the ASV in different samples from each environment subcategory. Using this methodology, we identified environments that potentially harbor novel Perkinsea organisms.

### Xcellidae distribution in the open ocean

In our dataset, we identified a notable presence of ASVs related to Xcellidae organisms, which were unexpectedly and widely distributed in the deep open ocean (below 200 depth meters). To further investigate this enigmatic group, we focused on the published dataset from the Malaspina 2010 Circumnavigation Expedition^58^. This dataset is unique because it includes a wide range of marine open ocean samples from surface to bathypelagic zone. For each sample, the V4 region of the SSU rRNA gene derived from DNA and RNA templates of the pico-eukaryotic fraction (between 0.2 and 3 µm) was sequenced. Samples were processed using DADA2^59^ and taxonomically annotated as described in Obiol et al.^60^. ASVs related to Perkinsea were extracted and subjected to the same phylogenetic analyses, as described above, to confirm their affiliations within the Perkinsea lineage.

Yet DNA-based sequences contribute to elucidating the Perkinsea community structure, these datasets also include metabolically inactive or dead cells. Hence, the rRNA/rDNA ratio can be used as a proxy for the ‘relative’ ribosomal activity of the identified Xcelliade clusters^58^. This ratio has been calculated based on the contribution of each ASV classified as Xcellidae to the RNA and DNA-derived samples.

## Results

### Phylogenetic classification of environmental Perkinsea sequences

Using a phylogenetic approach, we investigated the diversity and community structure of Perkinsea ASV sequences retrieved from the published EukBank dataset^37^.

We first conducted a phylogenetic analysis using a reference tree derived from an alignment of 1790 characters, which included representative alveolate groups and environmental sequences. This analysis successfully retrieved all major clusters described in the literature, including NAG01, Perkinsidae, Xcellidae, Parviluciferaceae, and environmental clusters (referred to as ‘Perkinsea environmental cluster 01–04’). These clusters exhibited robust node support in both maximum-likelihood and Bayesian inferences (TBE > 0.8 and PP > 0.90, Fig. S1). However, a few sequences, such as *M. nigrum* (MN721813.1 and MN721814.1) and *P. dinoexitiosum* (MZ663823.1 and MZ663830.1), displayed low branch support, underscoring the uncertainty in their phylogenetic placement as described in previous analyses^12,13^.

**Figure 1:**
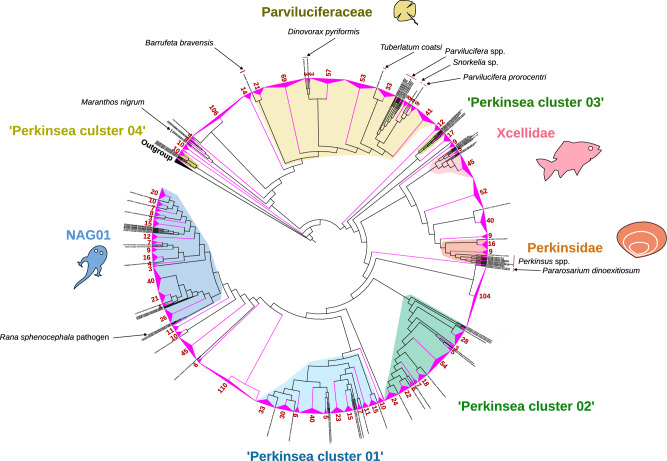
Phylogenetic placement of ASVs into the Perkinsea reference tree. The groups of ASVs were collapsed and highlighted in pink. The red numbers correspond to the number of ASVs in each collapsed cluster.

From the EukBank dataset, we extracted 1647 ASVs preliminary classified as Perkinsea group using a pipeline developed by the EukBank consortium (https://unieuk.net/). The taxonomic affiliation was checked using phylogenetic analysis with our reference dataset. Out of the 1647 ASVs, the phylogenetic analysis confirmed that 1568 ASVs were not chimeras and branched with known Perkinsea. Perkinsea ASVs represented 1,075,904 reads and were found across 4034 samples. These ASVs were placed throughout the phylogenetic tree, illustrating the extensive diversity retrieved within the Perkinsea lineage (Fig. 1). The LWR analysis provided high-confidence assignments for 67% of the ASVs, as they were placed within their respective branches with a certainty score (LWR) greater than 0.50 (Fig. S2). In total, 318 ASVs (~ 20.3%) were associated with Parviluciferaceae, 214 ASVs (~ 13.6%) with NAG01, 52 ASVs (~ 3.3%) with Xcellidae, 39 ASVs (~ 2.5%) with Perkinsidae and 5 ASVs (~ 0.3%) with *P. dinoexitiosum*. Additionally, some ASVs were classified into environmental clusters: ‘Perkinsea cluster 01’ (200 ASVs, ~ 12.7%), ‘Perkinsea cluster 02’ (172 ASVs, ~ 11.0%), ‘Perkinsea cluster 03’ (5 ASVs, ~ 0.3%) and ‘Perkinsea cluster 04’ (12 ASVs, ~ 0.8%). The remaining 551 ASVs (~ 35.2% of the ASVs) could not be assigned to any of the nine defined clades and were designated as ‘unclassified Perkinsea’ ASVs. No ASVs related to *M. nigrum* were detected in our analysis.

### Diversity and distribution of Perkinsea sequences

To investigate the diversity and distribution patterns of the identified Perkinsea, we classified the 4034 samples into three main environmental types: ‘Marine’ (2567 samples), ‘Land water’ (410 samples), and ‘Soil’ (1057 samples). Within these categories, we detected 1001 Perkinsea ASVs in ‘Marine’ samples, 601 ASVs in ‘Land water’, and 269 ASVs in ‘Soil’. The sequencing effort for each environment, represented by the percentage of Perkinsea ASVs out of the total number of reads, ranged from approximately 0.1% in both ‘Marine’ and ‘Soil’ environments to 2.4% in ‘Land water’. These findings are consistent with the results of Jobard et al.^25^.

**Figure 2:**
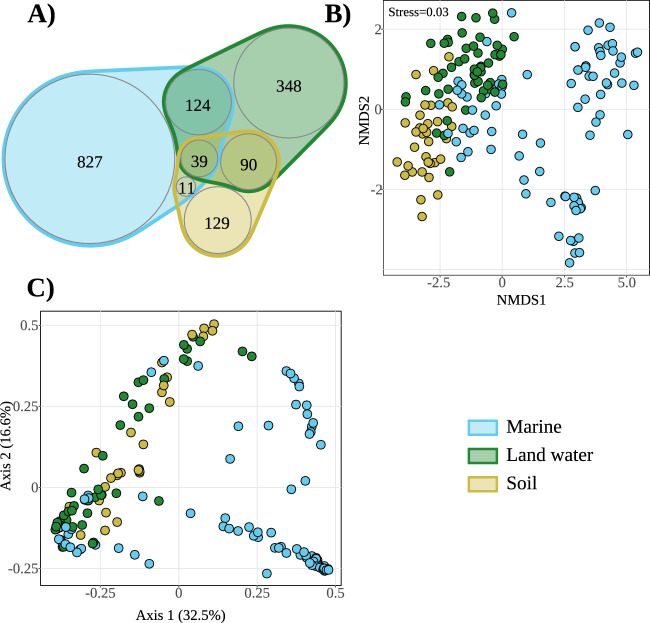
(**A**) Venn diagram of ASVs shared across environments. (**B**) Non-metric multi-dimensional scaling ordination plot (NMDS) based on Bray–Curtis distances, with 87 samples from ‘Marine’ environments, 53 from ‘Land water’ and 40 from ‘Soil’. (**C**) PCoA based on the phylogenetic distance of the samples.

Most ASVs exhibited habitat specificity, with 827 ASVs exclusively found in ‘Marine’ environments, 348 ASVs recovered exclusively from ‘Land water’ and 129 ASVs specific to ‘Soil’ (Fig. 2A). Only 39 ASVs were retrieved across all three categories. These shared ASVs were associated with NAG01 cluster (16 ASVs), ‘unclassified Perkinsea’ (13 ASVs), ‘Perkinsea cluster 02’ (8 ASVs) and ‘Perkinsea cluster 01’ (2 ASVs). ‘Marine’ environments shared 163 ASVs with ‘Land water’ and 50 ASVs with ‘Soil’. In contrast, ‘Land water’ and ‘Soil’ exhibited only 129 shared ASVs.

**Figure 3:**
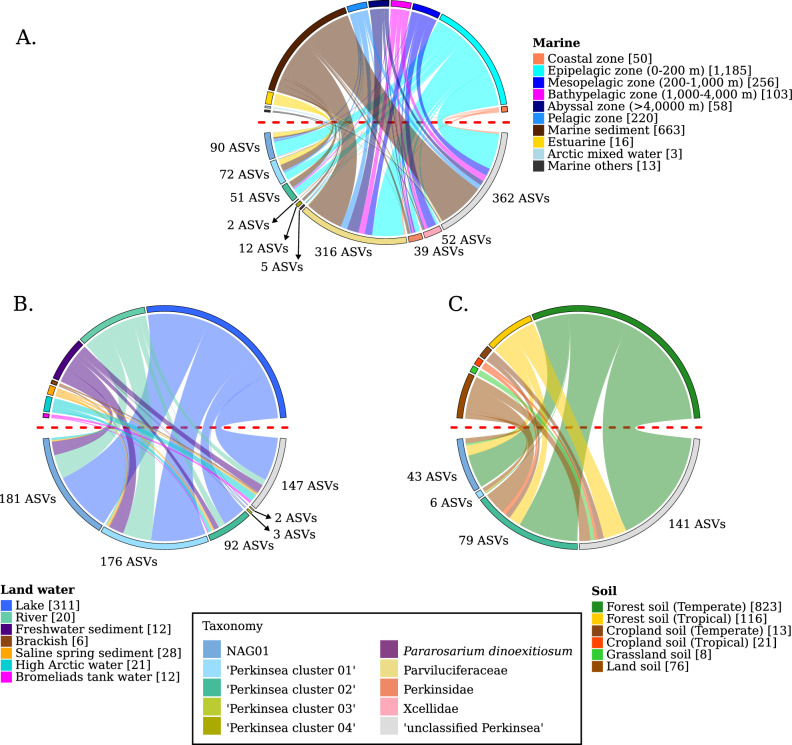
Distribution of Perkinsea ASVs into the different sub-environment categories. Each plot represents a specific main environment, such as Marine (**A**), Land water (**B**), and Soil (**C**). The description of each sub-category per environment is next to each plot. The width of the connectors is the number of ASVs corresponding to the specific taxonomic group. The taxonomic group description is in the lower boxed panel and applies to all plots.

The NMDS ordination plot based on the Bray–Curtis distance revealed a separation among ‘Marine’, ‘Land water’ and ‘Soil’ categories (Fig. 2B). According to ANOSIM analysis based on the Bray–Curtis distances (R:0.14, significance: 0.001, permutations: 999), the communities were significantly different between categories. A similar pattern was observed for the PCoA analysis based on the weighted UniFrac distance (Fig. 2C, R:0.29, significance: 0.001, permutations: 999). Our analysis also revealed a continuum between these ecological niches. ‘Soil’ and ‘Land water’ were more similar to each other than to ‘Marine’ samples, with the exception of ‘Estuarine’ and ‘Epipelagic zone’ samples that presented similarities with ‘Lake’ and ‘River’ samples (Figs. 2B, 3). Indeed, ‘Temperate forests’ shared 95 (78%) ASVs with ‘Lake’ while the ‘Epipelagic zone’ and ‘Estuarine’ shared 103 (20%) and 43 (78%) ASVs with ‘Lake’ environment, respectively (Table S1). Nevertheless, the communities Bray–Curtis and Weighted UniFrac distances showed to be significantly different (Pairwise ANOSIM *p* value < 0.05), with the exception of the Weighted UniFrac distance between ‘River’ and ‘Estuarine’ samples (*p* value  = 0.123, Table S2). The rest of Marine samples presented a very dissimilar community and can be separated into different groups corresponding to ‘Coastal zone’ samples positioned closely to ‘Marine sediments’ samples (sharing 76% of the ‘Coastal zone’ ASVs), separated from ‘Bathypelagic’, ‘Mesopelagic’ and ‘Abyssal zone’ samples. These last three environments share ~ 30% of the ASVs (Table S1). However, despite the relatively high number of shared ASVs, the communities differed significantly, including between ‘Epipelagic zone’ and ‘Marine sediment’, which shared 42% of the ASVs (*p* value  < 0.05, Table S2).

Alpha diversity index, including the Shannon index and phylogenetic diversity (PD), indicated that ‘Land water’ exhibited the highest diversity, with mean values of 1.1 and 1.3, respectively. In contrast, ‘Marine’ and ‘Soil’ samples showed lower mean alpha diversity and PD values, averaging around 0.4 (Fig. S4). Additionally, ‘Land water’ had the highest mean number of observed ASVs per sample (11 ASVs), followed by ‘Marine’ (2.3 ASVs per sample) and ‘Soil’ (2 ASVs per sample) (Fig. S4).

Regarding the Net Relatedness Index (NRI) analysis, higher indices were observed in marine water, particularly in the sub-category of ‘deep’ samples from the Abyssal zone, with a mean NRI of 1.18 and PD value of 0.69 (Fig. S4) This result suggests the presence of phylogenetically clustered ASVs in the ‘deep’ samples. However, rarefaction curves indicated that only the diversity of land water had been adequately sampled, as the curve reached a plateau, revealing that the Perkinsea diversity retrieved in ‘Marine’ and ‘Soil’ samples are still under-sampled (Fig. S5).

The Perkinsea ASVs revealed distinct affiliations for each taxonomic group. Indeed, phylogenetic analysis showed that ASVs from ‘Marine’ samples branched within all the identified groups, especially to ‘unclassified Perkinsea’ (362 ASVs, 36.2%) and Parviluciferaceae (316 ASVs, 31.6%). Additionally, 52 ASVs (5.2%) were affiliated with Xcellidae, and 39 (3.9%) ASVs were related to Perkinsidae clusters. A small number of ASVs (12, 1.2%) were associated with ‘Perkinsea cluster 04’, 2 (0.2%) to ‘Perkinsea cluster 03’ and 5 ASVs (0.5%) were related to *P. dinoexitiosum*. Surprisingly, a few ASVs from ‘Marine’ samples branched within clusters that are typically associated with freshwater environments, such as NAG01 (90 ASVs, 8.9%), ‘Perkinsea cluster 01’ (72 ASVs, 7.2%), and ‘Perkinsea cluster 02’ (51 ASVs, 5.1%).

To explore the ecological preferences of phylogenetic groups, particularly in marine samples, the category has been subdivided into sub-categories that represent different environments (Figs. 3 and S6). ASVs related to marine ‘unclassified Perkinsea’ were predominantly detected in marine sediments (228 ASVs) and the epipelagic zone (170 ASVs). A similar distribution was observed for Parviluciferaceae and Perkinsidae, with 182 and 12 ASVs detected in sediments and 151 and 23 ASVs in the epipelagic zone, respectively. Xcellidae ASVs were predominantly detected in the open ocean’s mesopelagic zone (42 ASVs) and bathypelagic zone (16 ASVs). ASVs related to ‘Perkinsea cluster 04’ were mainly observed in sediments (11 ASVs). Surprisingly, Perkinsidae (21 ASVs) and Parviluciferaceae (122 ASVs) were also detected in open ocean samples at significant depths, ranging from the ‘mesopelagic’ zone (200–1000 depth meters) to the ‘abyssal’ zone (below 4000 depth meters). The remaining detected groups in marine samples, including NAG01, ‘Perkinsea cluster 01–03’, were mainly detected in the epipelagic zone, estuarine areas, and sediments. ASVs related to *P. dinoexitiosum* were retrieved mainly from the epipelagic zone (4 ASVs), sediments (2 ASVs), and mesopelagic zone (1 ASV).

‘Land water’ samples were mainly represented by ASVs related to NAG01 (181 ASVs, 30.1% of the total ‘Land water’ ASVs), ‘Perkinsea cluster 01’ (174 ASVs, 29.3%), ‘Perkinsea cluster 02’ (92 ASVs, 15.3%), and ‘unclassified Perkinsea’ (147 ASVs, 24.5%). Additionally, ‘Perkinsea cluster 03’ (3 ASVs, 0.5%) and Parviluciferaceae (2 ASVs, 0.3%) were also detected in ‘Land water’ samples. ASVs related to NAG01 (268 ASVs), ‘Perkinsea cluster 01’ (267 ASVs), ‘Perkinsea cluster 02’ (63 ASVs) and ‘unclassified Perkinsea’ (157 ASVs) were mostly detected in lakes, rivers and sediment samples. Brackish water and Bromeliads tank samples were represented mainly by ASVs related to ‘unclassified Perkinsea’ (5 ASVs and 7, respectively) and ‘Perkinsea cluster 02’ (3 ASVs). In High Arctic water samples, ASVs related to ‘unclassified Perkinsea’ (20 ASVs) and ‘Perkinsea cluster 02’ (10 ASVs) were also retrieved.

Surprisingly, ‘Soil’ samples were represented by ASVs branched into as ‘unclassified Perkinsea’ (141 ASVs, 52.4% of the total ASVs observed in ‘Soil’ samples), ‘Perkinsea cluster 02’ (79 ASVs, 29.4%), NAG01 (43 ASVs, 16.0%) and ‘Perkinsea cluster 01’ (6 ASVs, 2.2%). ASVs related to ‘unclassified Perkinsea’ were detected in all ‘Soil’ subcategories. The next most abundant group in ASVs within ‘Soil’ samples group was ‘Perkinsea cluster 02’. NAG01 ASVs were retrieved in ‘Soil’ samples categorized as Temperate forests (33 ASVs), Tropical forests (10 ASVs), Temperate grassland (2 ASVs) and Temperate Land soils (5 ASVs). ‘Perkinsea cluster 01’ ASVs were detected in Temperate Land soil samples (4 ASVs) and Temperate and Tropical Forest samples (1 ASVs each).

A significant number of soil ASVs were exclusively retrieved from Temperate forest samples (96 ASVs), followed by Tropical forest samples (25 ASVs), and finally, Temperate Land soils (5 ASVs). However, these results may be influenced by the variable number of samples across each environment, which introduces potential biases. Nonetheless, we observed many ASVs shared between soil environments, even among contrasting ones such as Temperate and Tropical forests (14 ASVs). This indicates that potential Soil-dwelling Perkinsea organisms can adapt to diverse habitats.

### Potential novel Perkinsea taxa

**Figure 4:**
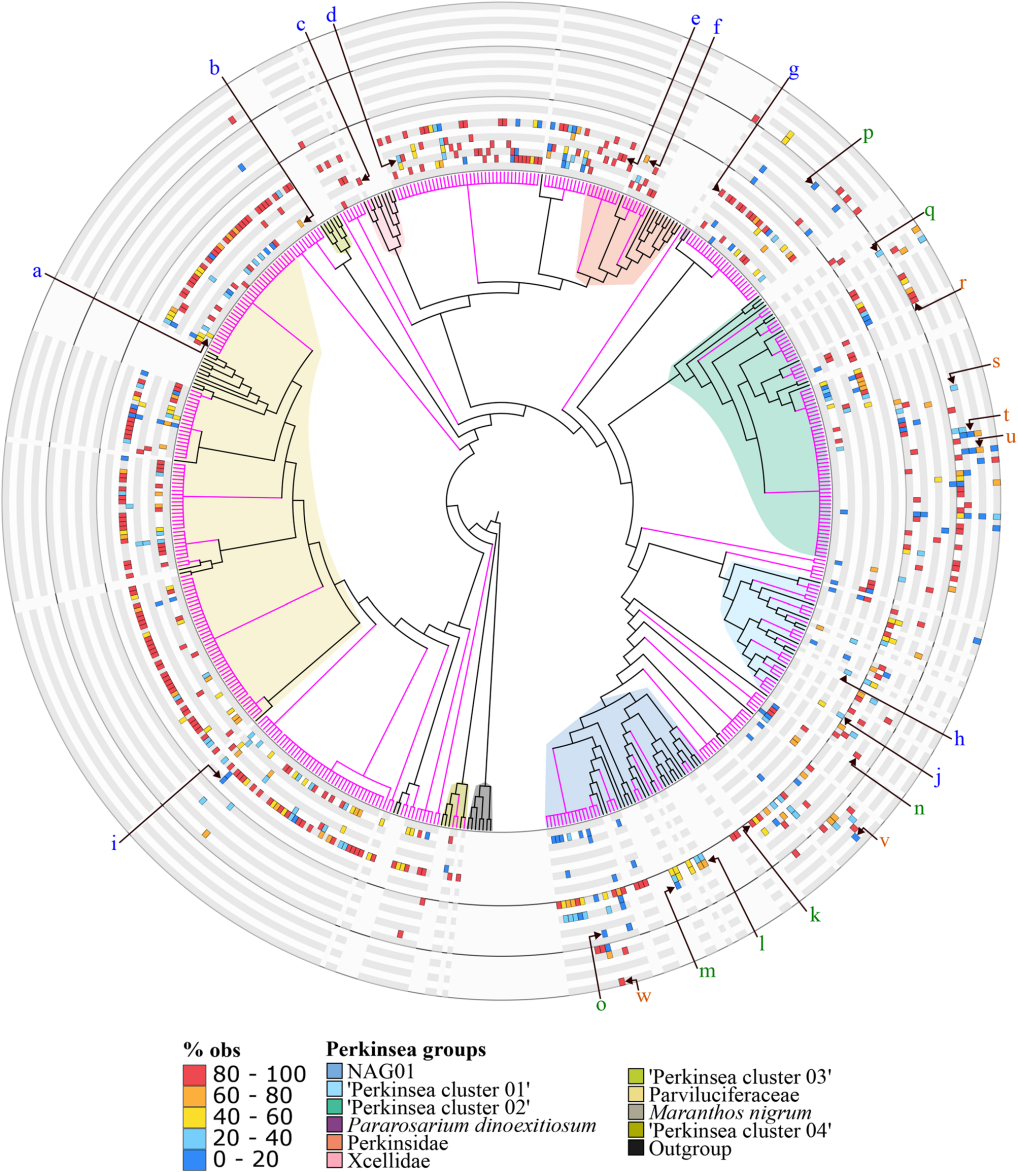
Phylogenetic placement of ‘Novel ASVs’ into the Perkinsea reference tree. The ASVs are highlighted in pink. The outer heat map circles show the sampling provenance of each ASVs. Each sub-circle is identified by a letter which corresponds to a sub-category: (**a**) ‘Coastal zone’, (**b**) ‘Epipelagic zone’ (0–200 depth meters), (**c**) ‘Mesopelagic zone’ (200–1000 depth meters), (**d**) ‘Bathypelagic zone’ (1000–4000 depth meters), (**e**) ‘Abyssal zone’ (below 4000 depth meters), (**f**) ‘Pelagic’ (include marine water samples from unspecified depth), (**g**) ‘Sediment’, (**h**) ‘Estuarine’, (**i**) ‘Artic mixed water’, (**j**) ‘Marine others’. (**k**) ‘Lakes’, (**l**) ‘Rivers’, (**m**) ‘Sediments’, (**n**) ‘Brackish’, (**o**) ‘Saline spring sediment’, (**p**) ‘High Arctic water’ and (**q**) ‘Bromeliads tank water’. (**r**) ‘Forest soil (Temperate)’, (**s**) ‘Forest soil (Tropical)’, (**t**) ‘Cropland soil (Temperate)’, (**u**) ‘Cropland soil (Tropical)’, (**v**) ‘Grassland soil (Temperate)’ and (**w**) ‘Land soil (Temperate)’. Color scales are detailed in the legend (left) (indicating the number of times the ASV was retrieved from the environment compared with the total number of observations of the ASV). The main different clusters described are indicated on the right of the legend box.

By applying specific criteria based on the LWR and the percentage of similarity (mean LWR < 0.69 and the mean % similarity < 94.8%), 428 ASVs potentially related to novel groups were identified (Fig. S7). These ‘novel’ ASVs branched widely across the reference tree (Fig. 4). Among them, 320 ASVs were detected in ‘Marine’ environments, 95 in ‘Land water’ and 50 in ‘Soil’ environments. The novel ASVs closely related to Parviluciferaceae were detected in marine sediments. ASVs related to Perkinsidae were mostly detected in the epipelagic and bathypelagic zones, except for one ASV detected in Abyssal zone samples (Fig. 4). Novel ASVs related to NAG01 were mainly retrieved from lakes and land water sediments (22 ASVs). Still, a significant number of these novel ASVs were also recovered from the marine epipelagic zone (7 ASVs) and the soil samples (3 ASVs in Temperate and 2 ASVs in Tropical Forest soil samples). ‘Perkinsea cluster 01’ novel ASVs were mostly detected in Land water samples, with a few observations in marine ‘epipelagic zone’ (3 ASVs) and sediment samples (3 ASVs). ‘Perkinsea cluster 02’ novel ASVs were retrieved in a wide range of samples, including 36 ASVs in soil and land water samples, 10 ASVs in marine samples, four ASVs in the Bathypelagic zone, and two ASVs in sediment samples. We can distinguish two groups among the ‘unclassified Perkinsea’ novel ASVs. Those closely branching to marine Perkinsea lineages were mostly detected in the Epipelagic zone and marine sediments, following the same patterns as the closely related defined lineages. However, those placed basal to NAG01, ‘Perkinsea cluster 01 to 03’ showed different distribution, with some ASVs solely retrieved in marine samples (17 ASVs) and others detected in both land waters and soil samples (18 ASVs). In total, 13 novel ASVs were detected within the three contrasted environments (‘Marine’, ‘Land water’ and ‘Soil’).

### Xcellidae distribution in open oceans

One noteworthy finding was that the ASVs related to Xcellidae were mostly detected in the Mesopelagic zone (between 200 and 1000 depth meters) and exhibited a global distribution across all the different oceans (Fig. S6). However, it is important to consider that the detected eDNA could originate from various sources, including free-living organisms, potentially infected host organisms, metabolically inactive or dead cells, or free DNA^28^. To investigate this hypothesis, we conducted a case study using the published Malaspina Expedition circumnavigation dataset. The expedition took place from 2010 to 2011 and involved sampling the tropical and subtropical regions of the Atlantic, Indian, and Pacific Oceans. This expedition is unique because (i) it covered vertical water column profiles spanning seven depth meters, ranging from the surface to 4000 depth meters and (ii) both rDNA and rRNA were used as templates for environmental sequencing. Using this dataset, we investigated the ‘relative’ ribosomal activity of Xcellidae in marine samples using DNA and RNA-derived sequencing^58^. We identified 90 ASVs related to Xcellidae. These ASVs contributed to less than 0.1% of the total reads in both DNA and RNA samples. We analyzed the rRNA/rDNA ratio, which can be a proxy for the ‘relative’ ribosomal activity. Our results highlight that, in most mesopelagic samples, the Xcellidae ASVs exhibited higher relative rRNA contributions than rDNA. This indicates a potential active ribosomal activity, suggesting the presence of putatively living organisms related to Xcellidae in the mesopelagic zone of the marine water column (Fig. S8).

## Discussion

In recent years, high-throughput sequencing or metabarcoding has been extensively used in eDNA surveys to investigate the diversity and distribution of protists^61^. The availability of large datasets, such as the EukBank project, which compiles 13,055 unique samples from a wide range of habitats worldwide (including marine, land water and soil), as well as the MetaPR2 dataset comprising over 5036 samples for the V4 region and 1166 samples for the V9 region of the SSU rRNA^62^, presents a unique and valuable opportunity to explore the community structure of Perkinsea in a wide range of ecosystems.

### Perkinsea distribution and potential colonization

Our study revealed that Perkinsea is a genetically diverse lineage, with 1568 ASVs identified and distributed worldwide. Marine environments were found to harbor the highest number of Perkinsea ASVs (1001 ASVs), although this could be influenced by the large number of marine samples (2537) compared to other environments (Fig. S5). The three main marine lineages of Perkinsea, namely Parviluciferaceae (318 ASVs), Xcellidae (52 ASVs), and Perkinsidae (39 ASVs), were particularly prominent. Their distribution exhibited a distinct latitudinal gradient. Xcellidae ASVs were mainly found in open ocean samples from middle latitudes, between 20 and 60 degrees, while Parviluciferaceae and ‘unclassified Perkinsea’ were more prevalent in high altitude samples, between 60 and 90 degrees (Fig. S6).

The presence of Parviluciferaceae ASVs at high latitudes, including polar samples, may be attributed to recurrent harmful algal blooms in the Arctic during the last years^63^. The Arctic region is known to host at least five families of noxious micro-algae, including the PSP-producer *Alexandrium catenella*^63^, which is susceptible to infection by *Parvilucifera sinerae* (Parviluciferae), previously described and isolated from the Mediterranean Sea^64^. These findings significantly expand the environmental range of Xcellidae and Parviluciferaceae organisms to previously unexplored marine regions such as open ocean and polar regions, respectively.

Furthermore, a gradient was observed within the marine samples throughout the water column. Specifically, samples from the Mesopelagic, Bathypelagic and Abyssal zones exhibited more similar community structures based on beta diversity (amount of differentiation between species/ASV communities) than samples from the epipelagic zone. Indeed, samples from the Mesopelagic and Bathypelagic zone presented not significance in the pairwise ANOSIM analysis taking into account the Weighted UniFrac distance (*p* value  = 0.193, Table S2). Interestingly, the diversity indices of the epipelagic zone were more similar to those of the ‘Land water’ samples, suggesting a potential differentiation of genetic signatures between deep ocean, surface or epipelagic zones.

In contrast to marine environments, Perkinsea contributed significantly to land water samples, accounting for approximately 2.7% of the total reads, with an average of 11 ASVs per sample and a maximum of 69 ASVs in the Sanabria Lake sample. These values were significantly higher than those observed in marine environments, indicating a greater presence and diversity of Perkinsea in land water ecosystems. Lakes and rivers being physically and chemically more heterogeneous than the ocean and lacking continuous geographical distribution, may favor local endemism and, therefore, a higher Perkinsea diversity^5^. Additionally, land water samples exhibited a high phylogenetic diversity (PD index) (Fig. S4), suggesting the coexistence of diverse Perkinsea lineages within the same sample. Previous studies have suggested that freshwater Perkinsea may act as parasites^27,30^. Indeed, Jobard et al. showed that freshwater Perkinsea in Lake Aydat and Bourget (France), during both summer and winter, were associated with *Sphaerocystis* (Chlorophyceae) and cyanobacteria filaments^25^. This highlights the potential parasitic lifestyle of Perkinsea and their putative role in influencing host-related phytoplankton dynamics in freshwater ecosystems. However, due to the vast genetic diversity retrieved in freshwater ecosystems and the lack of culture-based studies, it remains still enigmatic whether Perkinsea organisms can adopt other lifestyles. Further research is needed to unravel the ecological roles and functional characteristics of Perkinsea in freshwater ecosystems.

Although Perkinsea has primarily been associated with marine and freshwater environments^19,25,27,30^, our study identified 269 ASVs in soil samples (Fig. 2A). These ‘Soil’ Perkinsea ASVs branched into four different clusters, including ‘unclassified Perkinsea’ (141 ASVs), ‘Perkinsea cluster 02’ (79 ASVs), NAG01 (43 ASVs), and ‘Perkinsea cluster 01’ (6 ASVs) (Fig. 3). One hypothesis for these results is the potential windblown spread of freshwater Perkinsea, similar to what has been suggested for Dinophytes in Neotropical rainforest soil^65^. However, our analyses revealed that 129 ASVs were exclusively detected in soil samples and the communities and phylogenetic diversity is significantly different to land water (Fig. 2A and Table S2). Among the shared ASVs, only 14 ASVs were between samples from Tropical Forests (high precipitations with periods of floods) and Temperate Forest soils (wet and dry soils), highlighting different environmental preferences of soil Perkinsea (Table S1). Furthermore, NMDS analysis revealed a continuum between ‘Soil’ and ‘Land water’ samples based on their diversity composition, from ‘Land water’ to Temperate forests (Fig. 2B). Previous studies have shown that soil samples are rich in parasitic protists, which can influence animal diversity by limiting the population growth of locally abundant species^6^. On the other hand, the low contribution of these ASVs to the total number of reads in soil samples (< 0.1%) suggests that Perkinsea organisms may contribute to the soil seed bank, similar to the findings of Parviluciferaceae in marine coastal sediments^28,66–68^. Among soil subcategories, 86 ASVs were exclusively retrieved in temperate forest samples (Table S1), corresponding to only three locations in the Northern Hemisphere (Switzerland, Norway and Canada). The combination of these results with the low number of ASVs shared between soil environments (35 ASVs) may reflect the lower dispersal capabilities of soil Perkinsea organisms (e.g., by wind and animals)^69,70^ compared to aquatic protists. This is especially notable in marine environments where protists are likely to have higher dispersal due to high connectivity^70–72^. Therefore, future studies employing isolation and culture-based approaches are needed to confirm or refute the presence of Perkinsea in soil samples and to understand their role in these ecosystems.

During the diversification of Perkinsea, a transition from marine to freshwater environments occurred, as previously discussed by Chambouvet et al.^28^ and Bråte et al.^30^. This demonstrates the adaptability of the Perkinsea group to conquer and thrive in contrasting environments^30^. Our study also reveals that a transition to soil environments may be possible during the evolution of Perkinsea. The NMDS analysis based on beta diversity and the PCoA based on phylogenetic distance (Fig. 2B,C) showed distinct community compositions among the primary environments, indicating a potential transition from marine environments to freshwater and soil environments (Fig. S3). However, a more robust phylogenetic analysis with a complete 18S rRNA gene is needed to understand better how many times this succession happened during the evolution of Perkinsea.

### Perkinsea cryptic diversity

Our analysis uncovered many new cryptic taxonomic groups within the Perkinsea lineage. These groups can be categorized into two types: (i) sequences that could be assigned to known taxonomic groups (~ 27% of the total ASVs, ASVs classified as ‘Novel’ ASVs, LWR < 0.69 and the mean % similarity < 94.8%) and (ii) sequences that could not be assigned to any known taxonomic group (~ 35.1% of the total ASVs, ASVs classified as ‘unclassified Perkinsea’) (Figs. 4 and S7).

One group that exemplifies this cryptic diversity is the Perkinsidae, which currently includes seven described parasitic species known to infect bivalves and gastropods (Mollusks)^8^. Among them, the two infectious agents, *P. marinus* and *P. olseni,* are listed as notifiable diseases by the World Organization for Animal Health (O.I.E.)^8,10^. Interestingly, these Perkinsidae ASVs (16 ASVs) have been recovered in many marine ecosystems, spanning from the epipelagic zone to the abyssal zone (Fig. 4). This is noteworthy because most of the described species *Perkinsus* species have been isolated from coastal marine areas, where they infect commercially important host species^8^. Furthermore, the detection of two of these ASVs in marine sediment samples aligns with the known life cycle of described Perkinsus species, which involves a dormant stage in the sediment^28^. The presence of Perkinsidae ASVs in diverse marine environments suggests that the diversity and distribution of this group may be broader than previously recognized.

Similar results were observed for novel ASVs within the Parviluciferaceae, where all the ASVs were exclusively retrieved in marine waters, mostly from the Epipelagic zone and Sediments samples. This consistency is coherent with the known characteristics of described species within this group, parasites of dinoflagellates, a prominent and diverse group in terms of abundance and diversity in all oceans^11^. Environmental ASVs sequences were also recovered from marine sediments, which could be explained by the meroplanktonic life cycle of described Parviluciferaceae. In this life cycle, the activation of sporangia (benthic stage) and the release of infective free-living zoospores depend on host density^11,68,73^. A temporal series analysis is necessary to describe the life cycle of these potential novel organisms and how the environmental biotic or abiotic factors could activate the resting stages that govern their transition between the water column and sediments^67^.

Lastly, our analysis revealed the presence of six ASVs within the Xcellidae group that showed very low sequence similarity (< 0.6% of similarity) to known species, with only one ASV classified as novel (Fig. S7). Until now, only five species have been described within the Xcellidae family as parasites of 20 fish species belonging to five orders of teleosts^8^. This result potentially indicates the presence of sequences related to species that are currently not represented in public databases.

The detection of novel ASVs with low sequence similarity to known species within all known groups, including Perkinsidae, Parviluciferaceae, and Xcellidae, as well as those without any known taxonomic group, suggests that the diversity within Perkinsea may be much wider than previously thought. This is important because it raises intriguing questions regarding these putative organisms’ nature (e.g., symbiotic or free-living).

The Xcellidae group has remained enigmatic as it has not been previously reported in environmental surveys, except for only one ASV from the bathypelagic zone of the Gulf of California^15^. However, our analysis has provided, for the first time, the distribution of genetic signatures related to Xcellidae in open ocean samples, particularly in the mesopelagic zone (between 200 and 1000 depth zone), where these sequences dominate the Perkinsea dataset. The mesopelagic zone is a particular zone of the water column as it harbors the largest biomass of fish in open ocean^74^. Therefore, the presence of Xcellidae genetic signatures in this zone raises the possibility of these sequences being associated with infectious agents of fish. Indeed, Freeman et al.^15^ proposed that Xcellidae infections may occur through contact between fish and the benthos or fish-to-fish transmission. However, this study analyzed ASVs derived from DNA templates that could correspond to eDNA, free-living life stages released during their life cycle, and living or dead hosts. To overcome this issue, we analyzed the Malaspina dataset, which includes sequences derived from DNA and RNA templates. We showed that most of the Xcellidae ASVs were also recovered from the RNA template in the water column, suggesting the presence of potentially ‘ribosomally’ active life stages (Fig. S8). These findings provide valuable insights into the potential activity and ecological significance of putative organisms related to Xcellidae in the mesopelagic zone of the open ocean.

## Conclusions

Our study provides evidence that the Perkinsea lineage might be diverse and globally distributed. Marine environments harbor the highest number of Perkinsea ASVs, followed by land water samples. Surprisingly, we also identified a significant number of ASVs in soil samples, highlighting the need for further investigation to uncover the role of Perkinsea, especially in soil ecosystems. Numerous unclassified and novel ASVs within the Perkinsea lineage indicate potential cryptic diversity and underscore the importance of exploring and characterizing this lineage in greater detail.

Our analysis also reveals the dominance of Xcellidae within Perkinsea communities in the mesopelagic zone of the open Ocean, suggesting a potential association with fish hosts and/or the presence of a free-living infective life stage. These findings provide valuable insights into the potential ecological roles of Xcellidae in marine ecosystems, particularly in relation to fish hosts.

Using these results as the foundation of future work, the next challenge will be to confirm or refute if these genetic signatures are linked to specific organisms and/or represent novel taxa. Further research, including isolation and culturing efforts, as well as morphological and genomic characterization, will be essential to identify and understand the nature (e.g., symbiotic or free-living) and their ecological roles. *In fine*, further studies on Perkinsea diversity and their ecological roles are crucial for effective management and mitigation of the potential invasive and pathogenic impacts as it was described for specific species within this lineage. By gaining a comprehensive understanding of their diversity, distribution, and ecological interactions, we will be able to develop conservation strategies and mitigate potential environmental and economic consequences.

### Supplementary Information

Supplementary Figures.

Supplementary Tables.

## Data Availability

The data, metadata and scripts used for the analysis are available on the GitHub repository [https://github.com/sebametz/perkinsea_distribution]. The EukBank 18S V4 dataset is available on Zenodo repository (https://zenodo.org/records/7804946).
