# Supplementary material for: Global perspective of environmental distribution and diversity of Perkinsea (Alveolata) explored by a meta-analysis of eDNA surveys
Source: Sci Rep. 2023 Nov 17;13:20111. doi: 10.1038/s41598-023-47378-0 (PMC10656510; doi:10.1038/s41598-023-47378-0)
Supplement: Supplementary file 1 — Supplementary Figures. [file 41598_2023_47378_MOESM1_ESM.doc]

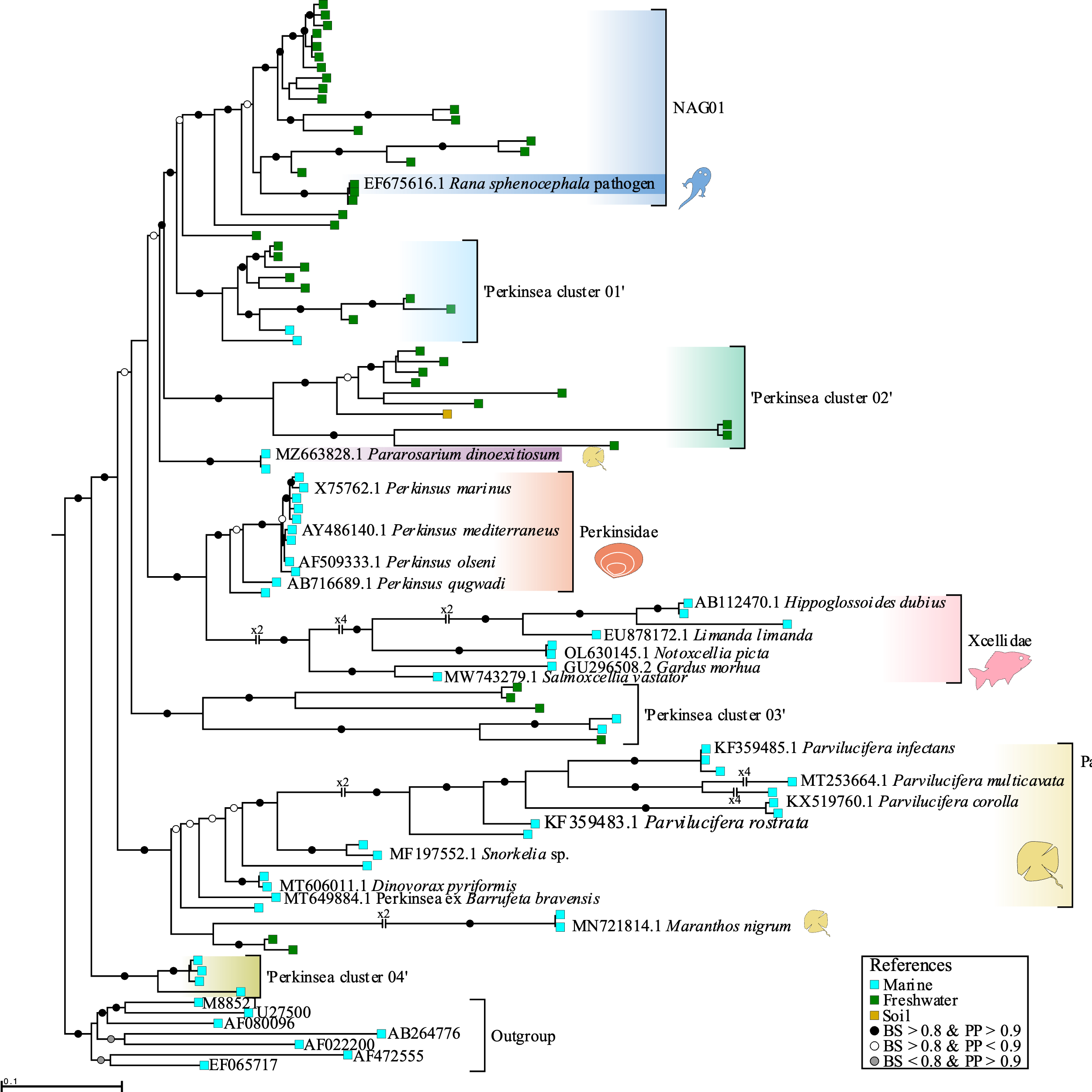


**Figure S1:** Maximum likelihood tree of the Perkinsea. The support for each defined taxonomic group was indicated with black dots when it was supported for RaxML and MrBayes (TBE>0.8 and PP > 0.9). Branches only supported by ML are highlighted with white dots and supported by MrBayes only with dots that are half white and half black. The color of the squad at the end of the branches corresponds to the environment from where the sequences were isolated. Reference sequences highlighted correspond to parasitic Perkinsea. The hosts are bivalves for the Perkinsidae, fishes for the Xcellidae, the dinoflagellates Maranthos nigrum and Pararosarium dinoexitiosum for the Parviluciferaceae and a tadpole for the sequence EF675616.1 (NAG01).


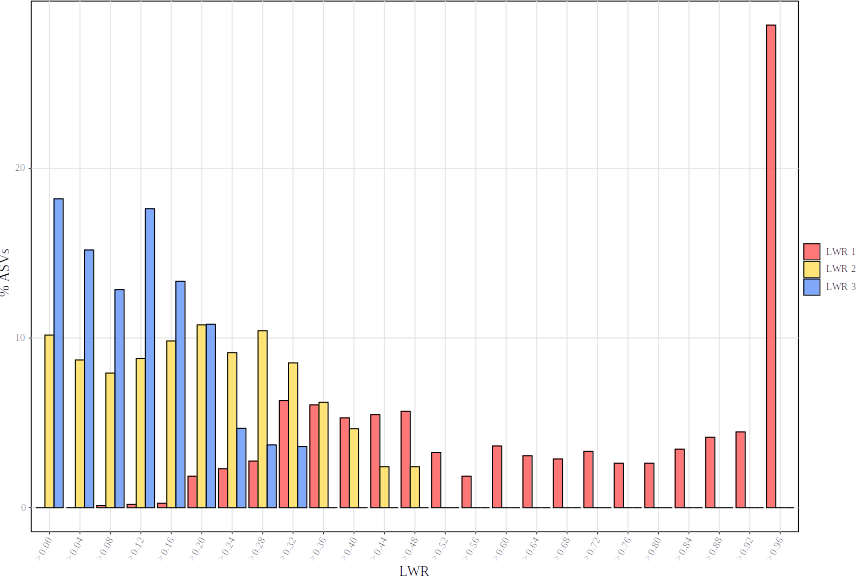


**Figure S2:** Histogram of the likelihood weight ratio (LWR) for the ASVs placement. The histogram shows the percentage of the first, second and third most likely placement.


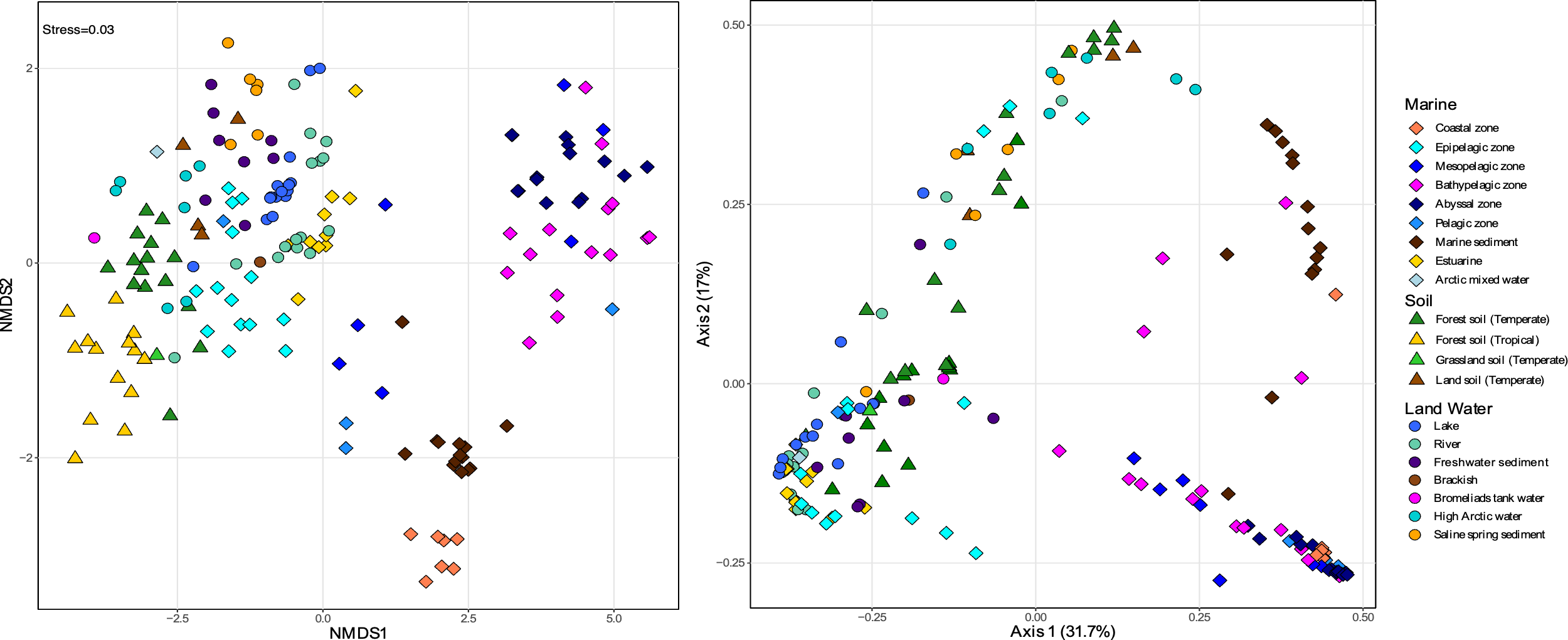


**Figure S3:** NMDS and PCoA with samples colored according to environment sub-categories classification.


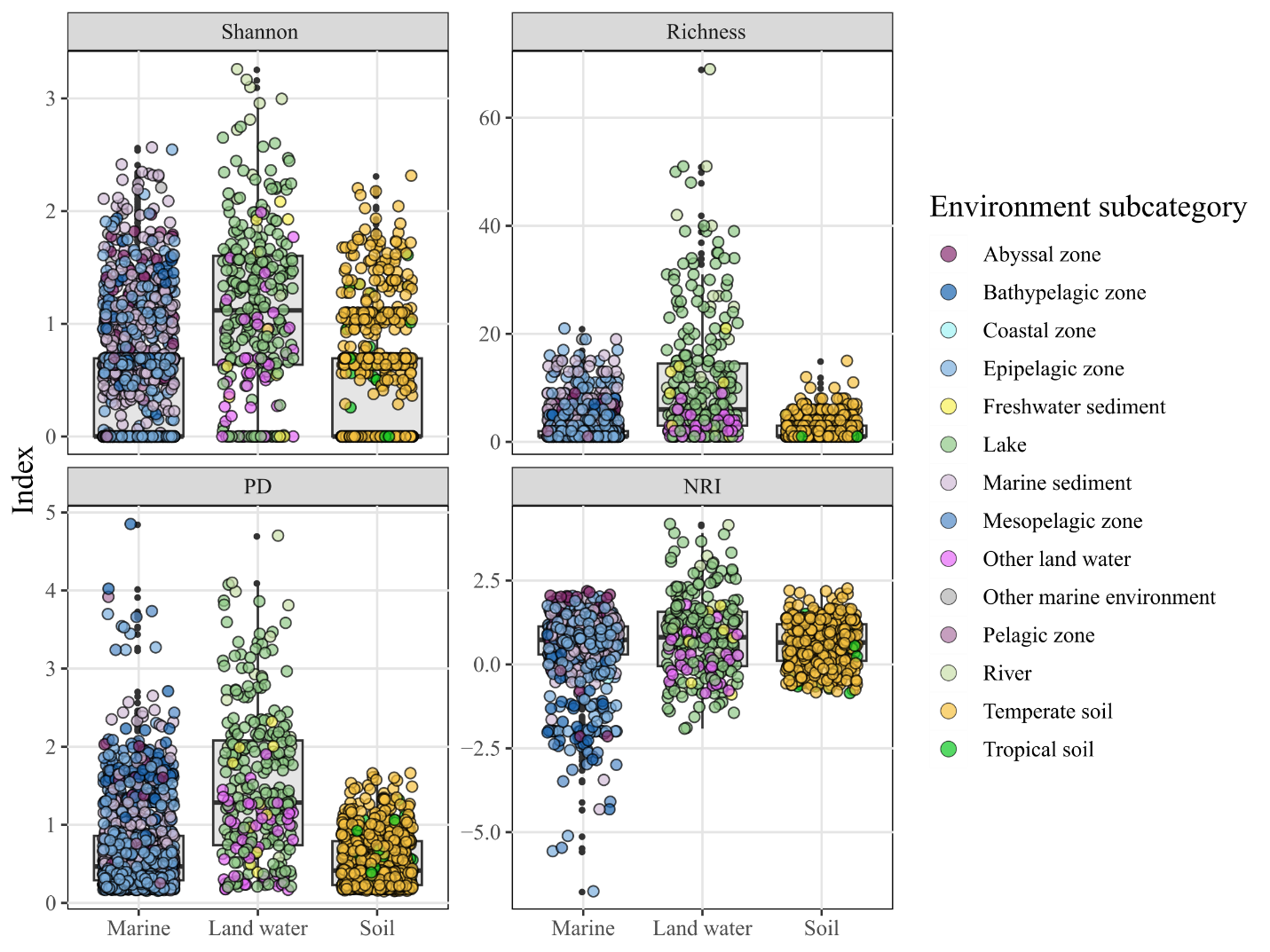


**Figure S4:** Diversity analysis of the Perkinsea samples. From top left to bottom right: Alpha diversity (Shannon). Richness or number of ASVs per sample, Faith’s phylogenetic diversity (PD) and Nearest Relative Index (NRI). The colors represent the different subcategories of the different environments.


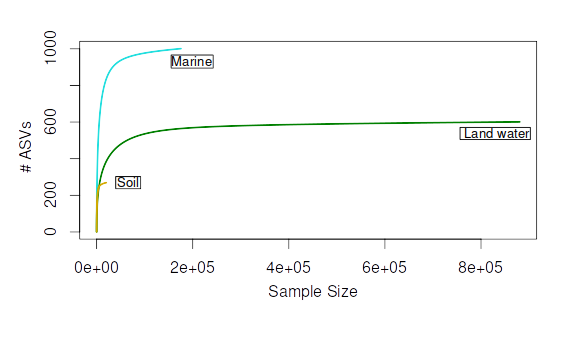


**Figure S5:** Rarefaction curves for the different environments studied.


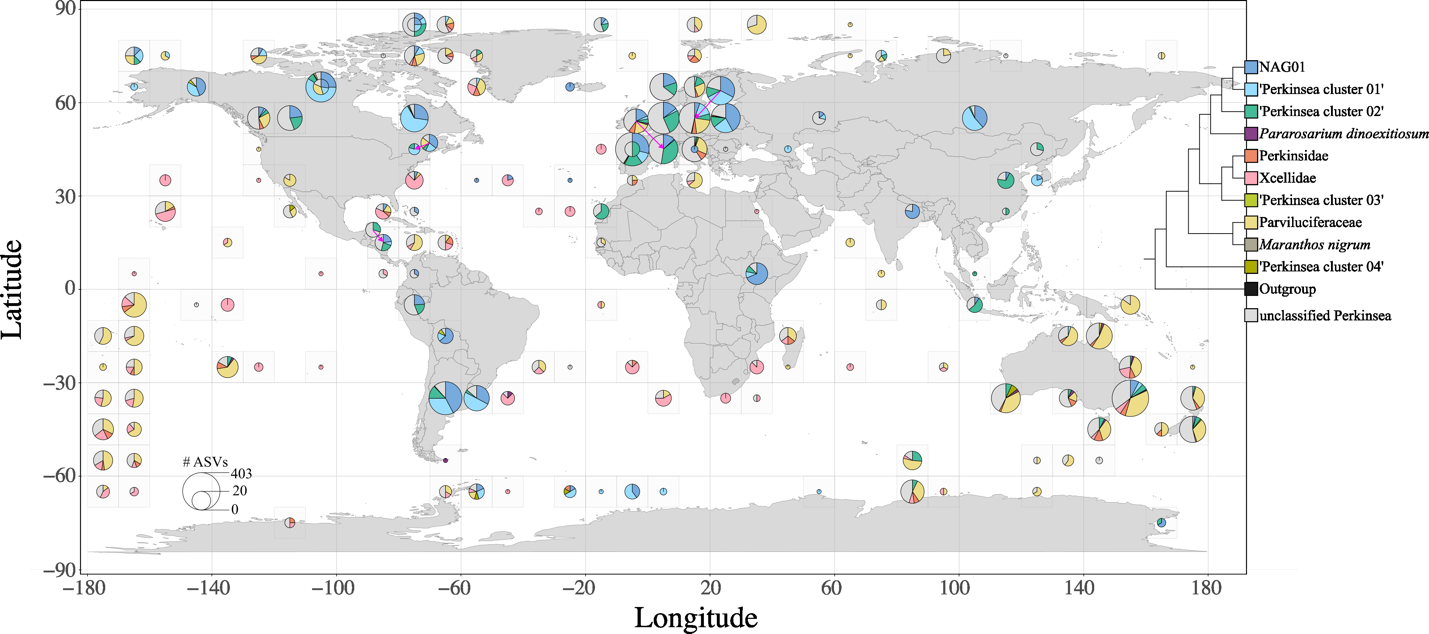


**Figure S6:** Global distribution of Perkinsea. The samples in a grid of 10x10 decimals in latitude and longitude were clustered together for better visualisation. The pie chart ratio represents the number of ASVs and the color of the proportion of ASVs related to each taxonomic group. The reference of the colors is a representation of the phylogenetic tree of the Perkinsea. The map was performed using R version 4.1.2 ([*https://www.r-project.org/*](https://www.r-project.org/)). The world map was obtained from the maps package version 3.4.1 ([*https://CRAN.R-project.org/package=maps*](https://CRAN.R-project.org/package=maps)), and the pie charts were plotted using ggplot2 version 3.4.1 ([*https://CRAN.R-project.org/package=ggplot2*](https://cran.r-project.org/package=ggplot2)).


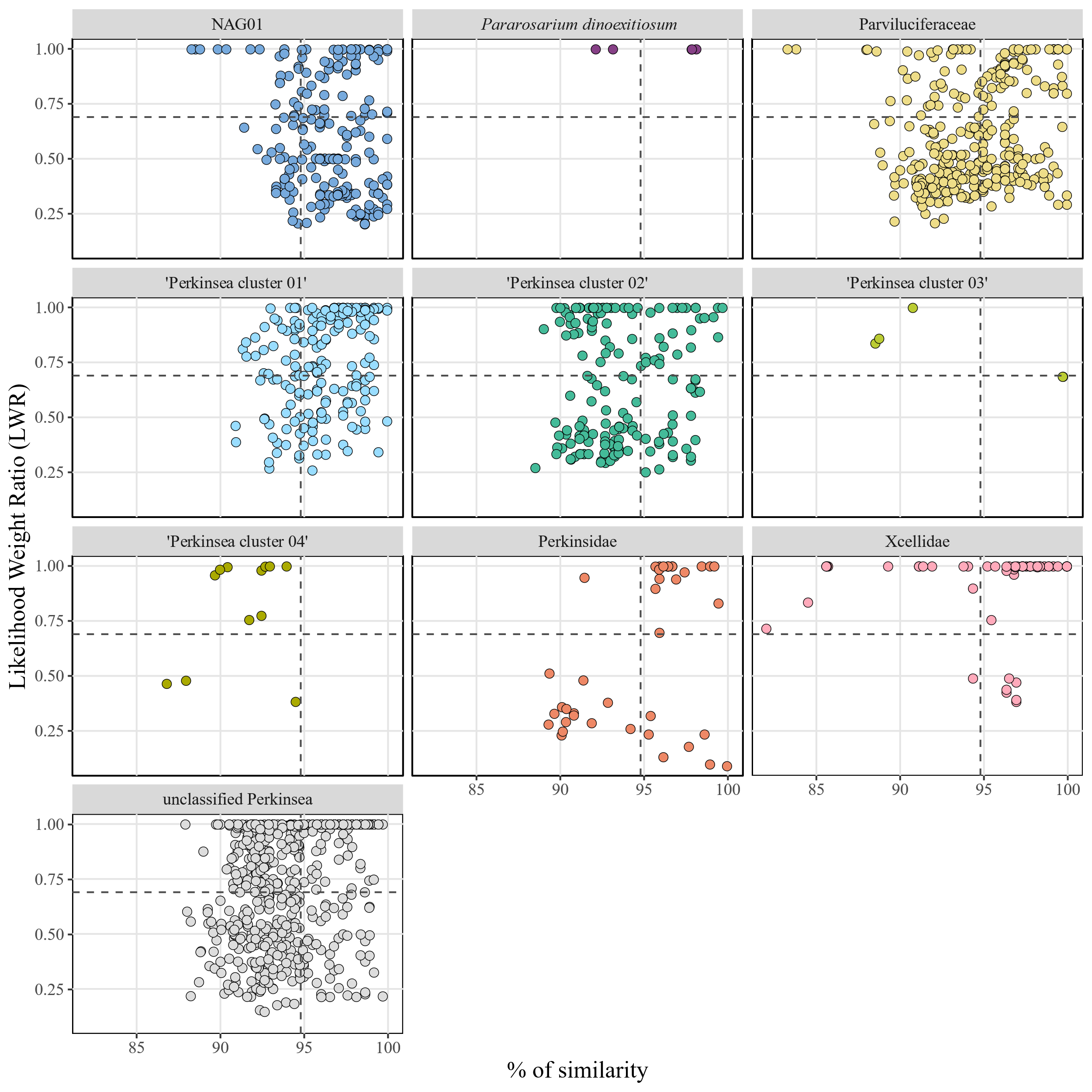
**Figure S7:** Biplot of the likelihood weight ratio (LWR) of the ASVs placement vs the % of similarity with the most similar sequence from the NCBI nr database. Broken lines are at the mean LWR and % of similarity, calculated by considering all the Perkinsea ASVs. ASVs with LWR and % of similarity lower than the mean, i.e., in the 3^rd^ quadrant of the biplot, were classified as novel.


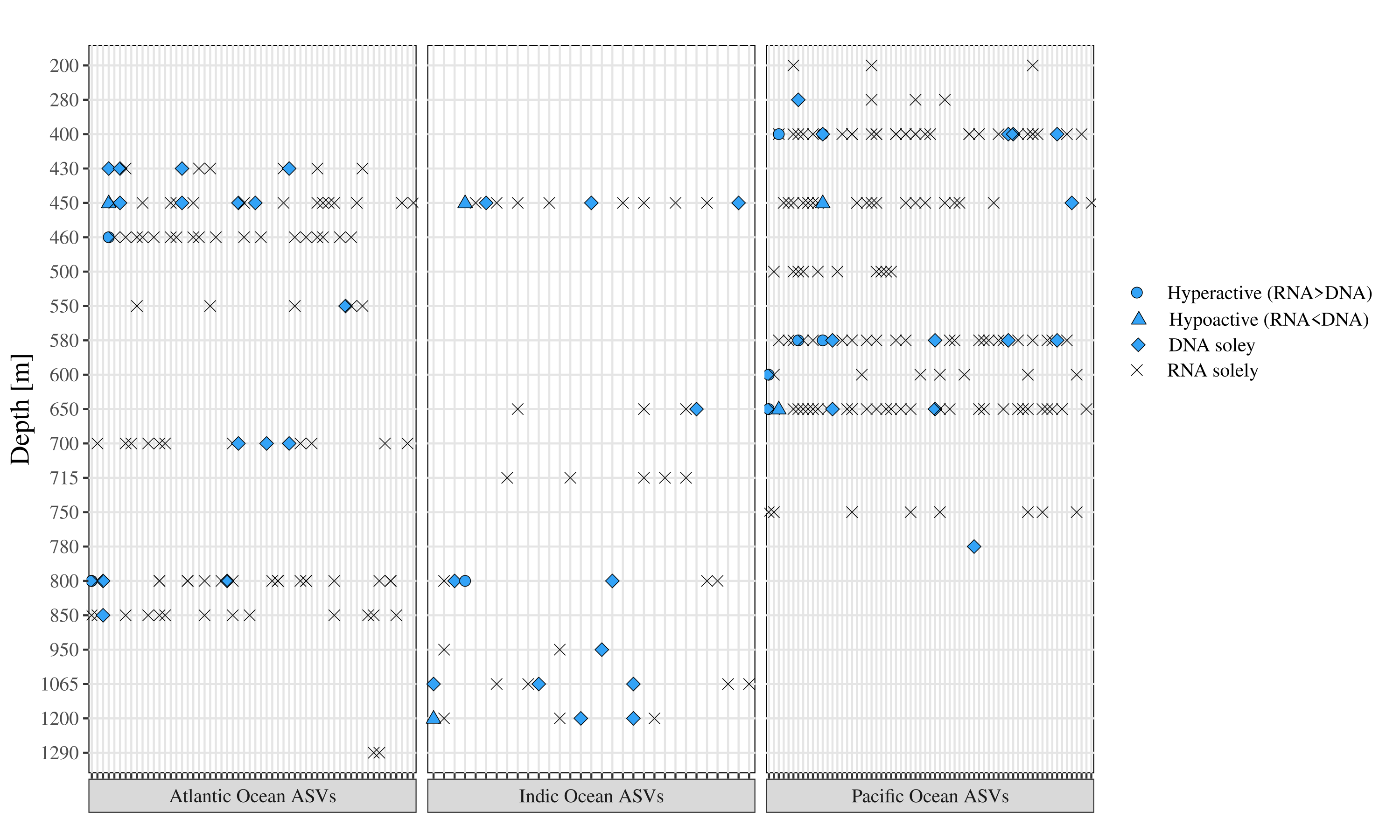
**Figure S8:** Xcellidae ASVs from the Malaspina dataset (Giner et al., 2020) and the RNA/DNA ratio observed at each sample depth at different stations across the ocean.
